# Supplementary figures and images for: PUPpy: a primer design pipeline for substrain-level microbial detection and absolute quantification
Source: mSphere. 2024 Jul 9;9(7):e00360-24. doi: 10.1128/msphere.00360-24 (PMC11288016; doi:10.1128/msphere.00360-24)

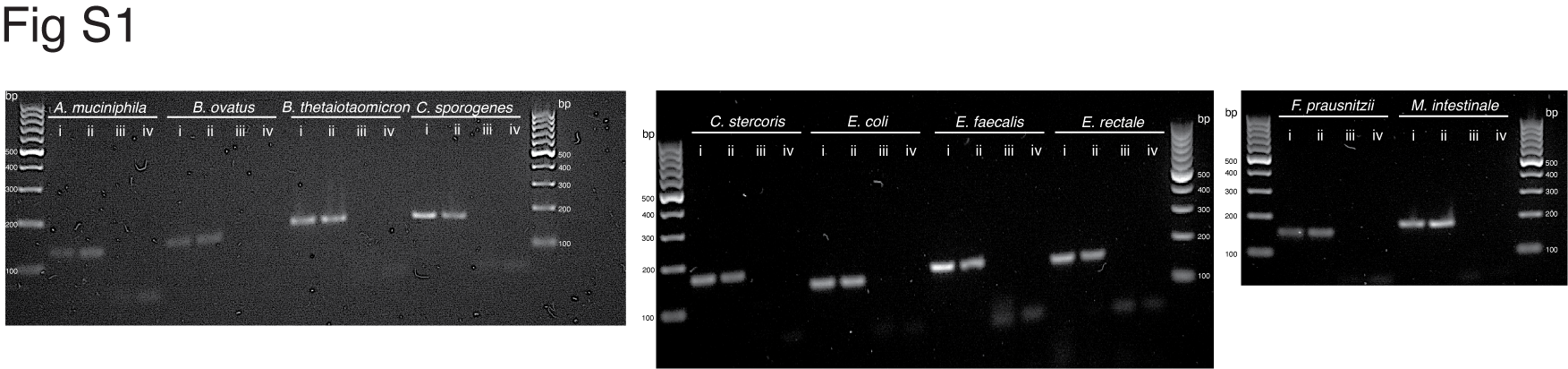

Supplement: Figure S1 — Supporting data for Fig. 2. [file msphere.00360-24-s0001.tif]

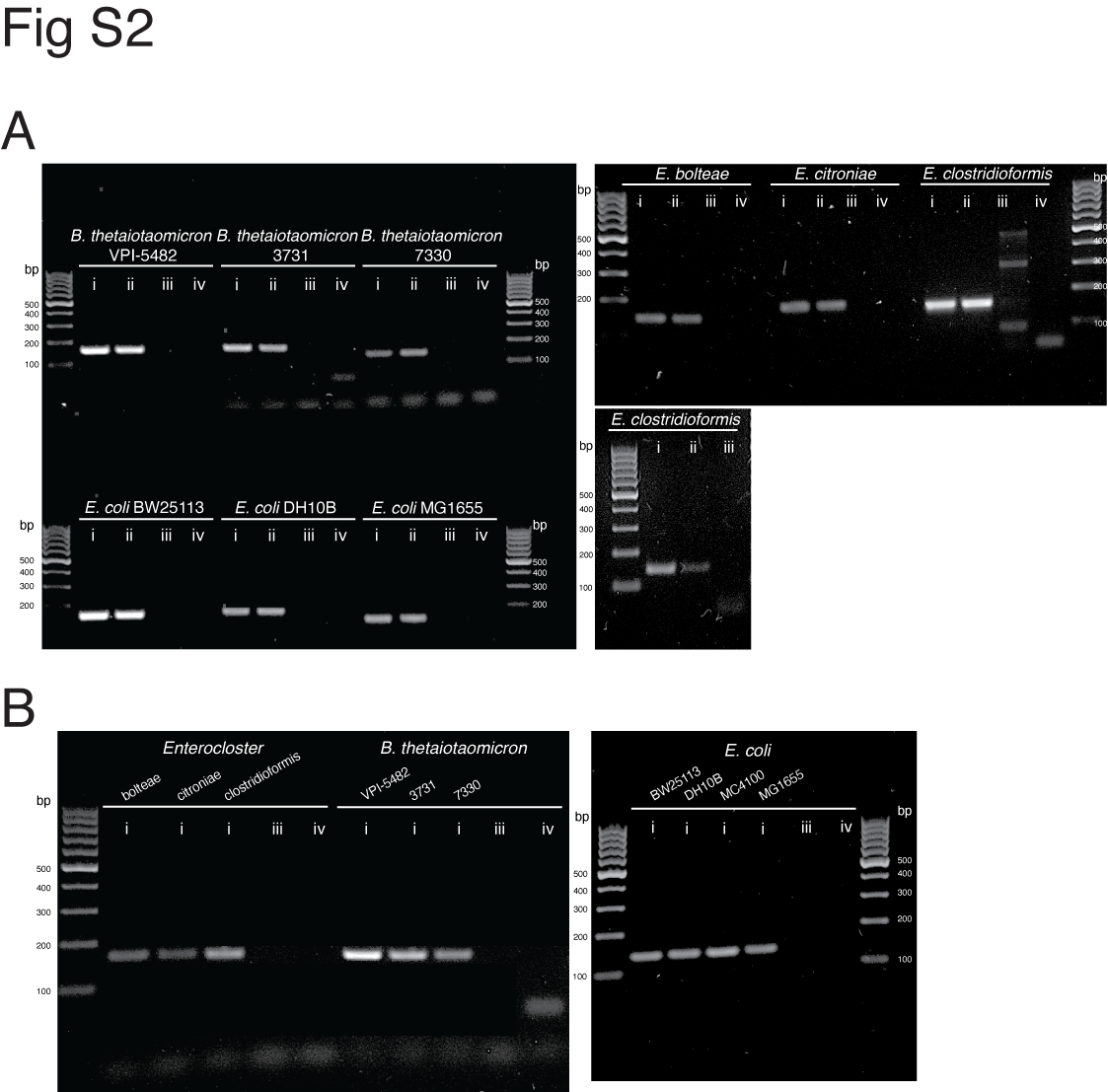

Supplement: Figure S2 — Supporting data for Fig. 3. [file msphere.00360-24-s0002.tif]

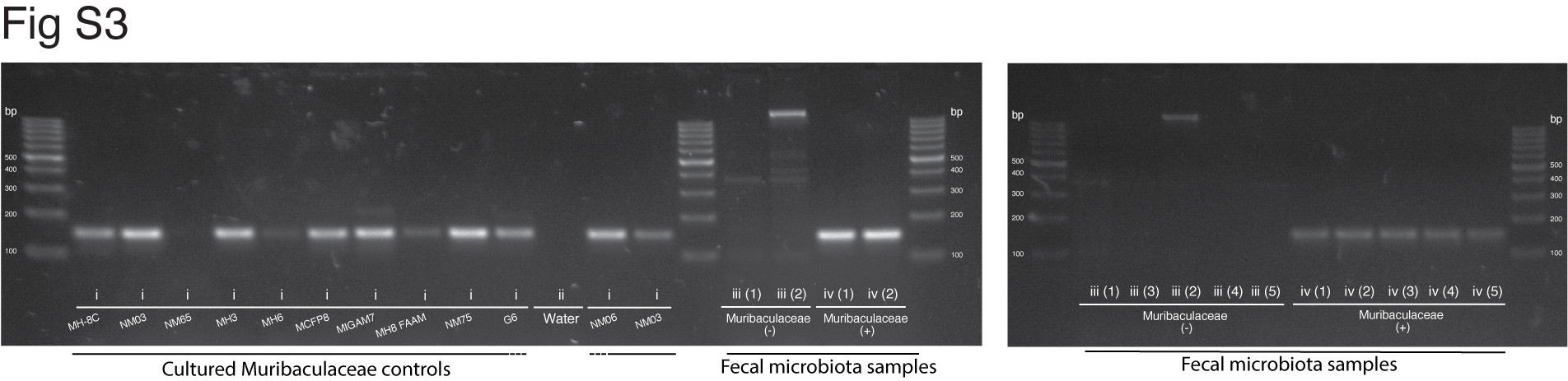

Supplement: Figure S3 — Supporting data for Fig. 3E. [file msphere.00360-24-s0003.tif]
