# Supplementary material for: PUPpy: a primer design pipeline for substrain-level microbial detection and absolute quantification
Source: mSphere. 2024 Jul 9;9(7):e00360-24. doi: 10.1128/msphere.00360-24 (PMC11288016; doi:10.1128/msphere.00360-24)
Supplement: Legends — Supplemental material legends. [file msphere.00360-24-s0005.docx]

**Supplemental Material Legends**

**Fig. S1**

PUPpy-designed microbe-specific primers selectively amplify all targets in the GUT community. Related to Fig. 2. Experimental validation of all microbe-specific primers by polymerase chain reaction (PCR) and gel electrophoresis, following the conditions outlined in Fig. 2C. All 10 microbe-specific primers selectively amplify their respective target, showing no unintended amplification. Faint primer dimers are visible in *A. muciniphila*, *B. thetaiotaomicron*, *C. sporogenes*, *E. faecalis*, and *E. rectale*.

**Fig. S2**

PUPpy-designed microbe- and group-specific primers selectively amplify the respective targets in the SSS community. Related to Fig. 3. (A) Experimental validation of all microbe-specific primers by polymerase chain reaction (PCR) and gel electrophoresis, following the conditions outlined in Fig. 3C. The initial validation of *E. clostridioformis* specific primers (top right gel) against the (-) pool without the intended target (condition *iii*) showed unspecific amplification at different sizes than the expected amplicon. Decreasing the extension time to 30 seconds (instead of 1 minute) prevented unspecific amplification, as seen in the small gel. The water control was not repeated because condition iii also acts as a negative control. (B) Experimental validation of group-specific primers by PCR, following the conditions outlined in (A). The gels were imaged on distinct runs. All group-specific primers selectively amplified their respective targets without unintended or unspecific amplification.

**Fig. S3**

PUPpy-designed Muribaculaceae-specific primers selectively amplify Muribaculaceae members in a complex microbial community. Related to Fig. 3E and 3F. Experimental validation of Muribaculaceae-specific primers by polymerase chain reaction (PCR) and gel electrophoresis, following the conditions outlined in Fig. 3E. The 2 PCR gel images were taken in distinct instances. The Muribaculaceae-specific primer mix amplified 10 different Muribaculaceae members, including strains that were not originally included in the PUPpy input for specificity checks (See Table S1 and S2). *M. intestinale* NM03 was run twice. *M. intestinale* NM65 was the only member not to be amplified, likely due to low DNA content. In the fecal samples, the Muribaculaceae-specific primer mix accurately discriminated the absence (*iii*) and presence (*iv*) of Muribaculaceae members in a complex microbial community, while also enabling strain-level resolution (*i*). The numbers in brackets for the fecal samples indicate biological replicates.

**Fig. S4**

Microbial quantification using only strain-informative shotgun sequencing reads under-estimates abundance of strains and substrains. Related to Figure 4C. Microbial quantification of SSS community members was estimated using shotgun sequencing (left panel) and ddPCR (right panel). In shotgun sequencing, relative abundance of the total reads was quantified using Bracken, Kraken2 at the Lowest Common Ancestor (LCA) level, and Kraken 2 while only considering unique reads, which are reads that map unambiguously to individual members of the community.

**Table legends**

**Table S1. Metadata of all microbes used for the design and validation of PUPpy-designed taxon-specific primers.** Microbes with “NA” noted under the growth conditions columns (last 4) were not cultured but only used for *in silico* primer design. Media ingredients and recipes for culture conditions can be found in Table S3.

**Table S2. Primers sequences and essential parameters for taxon-specific primers designed and validated in this study.** The column ‘target organisms’ exclusively refers to the specificity within the community for which primers were designed and does not necessarily extend to other microbial communities.

**Table S3. Media ingredients and recipes used to culture microbes in this study.** Details on culture conditions and media for each microbe can be found in Table S1.
